# Combined exposure to PM_2.5_ and PM_10_ in reductions of physiological development among preterm birth: a retrospective study from 2014 to 2017 in China

**DOI:** 10.3389/fpubh.2023.1146283

**Published:** 2023-07-26

**Authors:** Bo Hu, Jie Tang, Guangtao Xu, Dongliang Shao, Huafei Huang, Jintong Li, Huan Chen, Jie Chen, Liangjin Zhu, Shipiao Chen, Bin Shen, Limin Jin, Long Xu

**Affiliations:** ^1^Department of Preventive Medicine, Forensic and Pathology Laboratory, Institute of Forensic Science, College of Medicine, Jiaxing University, Jiaxing, Zhejiang, China; ^2^Department of Pathology and Key-Innovative Discipline Molecular Diagnostics, Jiaxing Hospital of Traditional Chinese Medicine, Jiaxing University, Jiaxing, Zhejiang, China; ^3^Department of Neonatal Intensive Care Unit, Jiaxing Maternity and Child Health Care Hospital, Jiaxing University, Jiaxing, Zhejiang, China

**Keywords:** PM_2.5_, PM_10_, birth outcomes, low birth weight, preterm birth

## Abstract

**Background:**

Preterm birth (PTB) has been linked with ambient particulate matter (PM) exposure. However, data are limited between physiological development of PTB and PM exposure.

**Methods:**

Trimester and season-specific PM exposure including PM_2.5_ and PM_10_ was collected from Jiaxing between January 2014 and December 2017. Information about parents and 3,054 PTB (gestational age < 37 weeks) outcomes such as weight (g), head circumference (cm), chest circumference (cm), height (cm) and Apgar 5 score were obtained from birth records. We used generalized linear models to assess the relationship between PTB physiological developmental indices and PM_2.5_, PM_10_ and their combined exposures. A binary logistic regression model was performed to assess the association between exposures and low birth weight (LBW, < 2,500 g).

**Results:**

Results showed that there were 75.5% of low birth weight (LBW) infants in PTB. Decreased PM_2.5_ and PM_10_ levels were found in Jiaxing from 2014 to 2017, with a higher PM_10_ level than PM_2.5_ each year. During the entire pregnancy, the highest median concentration of PM_2.5_ and PM_10_ was in winter (61.65 ± 0.24 vs. 91.65 ± 0.29 μg/m^3^) followed by autumn, spring and summer, with statistical differences in trimester-specific stages. After adjusting for several potential factors, we found a 10 μg/m^3^ increase in joint exposure of PM_2.5_ and PM_10_ during the entire pregnancy associated with reduced 0.02 week (95%CI: −0.05, −0.01) in gestational age, 7.9 g (95%CI: −13.71, −2.28) in birth weight, 0.8 cm in height (95%CI: −0.16, −0.02), 0.05 cm (95%CI: −0.08, − 0.01) in head circumference, and 0.3 (95%CI: −0.04, −0.02) in Apgar 5 score, except for the chest circumference. Trimester-specific exposure of PM_2.5_ and PM_10_ sometimes showed an opposite effect on Additionally, PM_2.5_ (OR = 1.37, 95%CI: 1.11, 1.68) was correlated with LBW.

**Conclusion:**

Findings in this study suggest a combined impact of fine particulate matter exposure on neonatal development, which adds to the current understanding of PTB risk and health.

## 1. Introduction

Preterm birth (PTB) is defined as a live birth less than 37 completed weeks of gestation and has become a global health problem. An estimated 15 million PTB infants in 2014, and over 1 million children die each year because of PTB complications, which account for 16% of all deaths and 35% of neonate deaths in 2019 (1). Research shows that in China, the incidence of PTB has ascended in the past three decades with a range from 5.36% in 1990–1994 to 7.04% in 2015–2016 (2). Due to the immature organs on structure and function, PTB increases short-term or long-term adverse impacts, such as poor growth, acute morbidity, respiratory illnesses, neurocognitive disorders, or chronic diseases in adulthood, leading to heavy social and economic burdens (3).

The causes of PTB are complex. It can be directly launched by multiple mechanisms such as hormonal disorders, intrauterine infection and inflammation, uteroplacental ischemia and hemorrhage, and other biological processes (4). The inducing factors, like uterine overdistension and cervical insufficiency, or epidemiological risk factors such as ethnicity, low socio-economic status, maternal weight, smoking, and periodontal status have been involved in PTB development (5). There is a wealth of population-based studies demonstrating that ambient air pollutants can increase PTB risk in the last two decades, even though the evidence of causal relationship is insufficient. In regards to specific pollutants, particulates seem the most important for infant deaths, of which PM_2.5_ and PM_10_ are mostly concentrated (6). Though particulate matter (PM) exposure has been considered as an important risk factor, but the evidence is variable. Qian et al. (7) showed that PM_2.5_, PM_10_, CO, and O_3_ were associated with increases in the risk of PTB, while no critical exposure windows were identified consistently. Most studies have focused on birth weight, indicating that higher exposure to PM_10_, NO_X_, SO_2_ and VOC is associated with reduced birth weight (8), and elevated PM_2.5_ concentration over the entire pregnancy and in the first trimester inversely correlates with low birth weight (LBW) (9). Several studies conclude inverse or null associations of PM_2.5_ with small for gestational age or term LBW (10, 11). Therefore, studies are essential to investigate this causal relationship of air pollutant exposure with PTB, as well as associations between combined exposure and neonatal developmental outcomes. Thus we aimed to monitor the PM_2.5_ and PM_10_ in Jiaxing, a city in eastern China, and examine the relationship between exposures and birth outcomes of physiological development in PTB. This study could provide a further understanding of neonatal birth outcomes and PTB risk following PM exposure.

## 2. Methods

### 2.1. Population and birth outcomes

This was a retrospective study, and subjects in a fixed hospital affiliated to Jiaxing University were enrolled between January 2014 and December 2017, and Jiaxing University stood in the near center part of the Jiaxing city. The inclusion criteria mainly included: (i) gestational age < 37 weeks, and LBW was less than 2,500 g; (ii) mothers lived in Jiaxing city at least for 1 year or above. The exclusion criteria were listed: (i) Parental races were Han, and there was no consanguineous marriage; (ii) No genetic diseases; (iii) Denied medicine or food allergic history; (iv) There were no records of cardiovascular and cerebrovascular diseases, lung, liver, kidney, endocrine and other important organ disorders. A total of 3,054 PTB infants were recruited. Clinic records including general information such as maternal education, father education, parental smoking, etc., as well as clinical delivery information such as placenta abnormality, maternal history and delivery times were obtained. PTB physiological development indices including weight (g), head circumference (cm), chest circumference (cm), height (cm), and Apgar 5 score were collected from the electronic medical records system. This study was approved by the Human Ethical Committee of Jiaxing University Medical College (JUMC-IRB-2019).

### 2.2. PM_2.5_ and PM_10_ data monitoring

The sampling area (30°77′ N, 120°76′ E) was from Jiaxing city, which located in the northern part of Zhejiang province, China. Air pollution indicators including PM_2.5_ and PM_10_ exposure were collected from the National Environmental Monitoring Center.^1^ In this study, the length of exposure for participants was divided based on seasons (spring, summer, autumn and winter) and pregnancy time (first trimester, second trimester, and the last trimester).

### 2.3. Statistical analyses

Continuous variables were expressed as mean ± standard deviation (SD), and categorical variables were described as the number of cases (%). PM_2.5_ and PM_10_ levels were skew distributed and presented as median ± SEM (standard error of the mean; min~max), and the comparison between multiple groups was performed by the Kruskal-Wallis H test with a further comparison by *post hoc* test. Generalized linear models were made to explore the relationship between PTB physiological indicators and PM_2.5_, PM_10_ and joint exposure. With regards to the joint exposure, we created a new variable calculated by the product of PM_2.5_ and PM_10_ (PM_2.5_*PM_10_). Studies have shown that interaction on a multiplicative scale or an additive scale indicates that the joint effect of the two exposures is larger/smaller than the product or sum of their individual effects (12). In the models, birth indicators were dependent variables, and the subject number was placed as the main variable in the random effect model, with confounding factors adjusted. These confounders were mother education level (1 = Primary school or below; 2 = Middle school; 3 = High school; 4 = college or above), mother smoking (1 = Yes; 2 = No), father smoking (1 = Yes; 2 = No), maternal age (1 ≤ 18 years; 2 = 19~35 years; 3 > 35 years), mother alcohol consumption (1 = Yes; 2 = No), delivery times and gestational age (w), which were considered because they have been commonly verified to be associated with preterm birth or neonatal development (13). Factors and covariates were included in the main effects of the fixed model, with the model effect test to determine whether it was statistically significant (β; 95% confidence). The beta coefficient indicated a unit change in birth indicators caused by a 1 μg/m^3^ increase in exposures. The binary logistic regression model was performed to assess the association between exposures and LBW (categorized by less than 2,500 g in all PTB) as the dependent variable, and models were adjusted for confounding factors of mother education level, mother smoking, father smoking, mother alcohol consumption, neonate gender, gestation, maternal age, delivery times and delivery way. An OR value represented the risk of a unit change in LBW for an increase of 1 μg/m^3^ of exposure level. Microsoft Excel, Graphpad Prism5 and SPSS22.0 software were applied to manage and analyze the data. A *p*-value <0.05 or 0.01 in a two-tailed test was considered to be statistically significant.

## 3. Results

### 3.1. General characteristics of the study population

There were 3,054 childbearing women with PTB enrolled from 2014 to 2017 (Table 1). The mean maternal age was 28.7 (±5.1) years. The mean gestation week was 34.2 (±1.6), and male neonates accounted for 55.7%. Maternal education was more frequent in college or above. The frequency of the mother smoking was less than that of the father. Pregnancy-related clinical records were presented. The PTB physiological indicators of birth weight, head circumference, chest circumference, height and Apgar 5 score were shown in the table, with a proportion of 75.5% low birth weight (LBW) infants.

**Table 1:** Characteristics of the study population and physiological indicators (*N* = 3,054).

Characteristics	*n* (%)^a^	Mean ± SD (Min–Max)
**Neonate gender**		
Male	1,700 (55.7)	
Female	1,350 (44.2)	
Maternal age (years)		28.7 ± 5.1 (14–47)
≤18	41 (1.3)	
19~35	2,668 (87.4)	
>35	324 (10.6)	
Gestational age (Weeks)^b^		34.2 ± 1.6 (28–36)
**Mother education level**		
Primary school or below	76 (2.5)	
Middle school	131 (4.3)	
High	559 (18.3)	
College or above	2,269 (74.3)	
Mother smoking	25 (0.81)	
Father smoking	2,103 (68.9)	
Mother alcohol consumption	159 (5.2)	
**Delivery mode**		
Eutocia	1,172 (38.4)	
Cesarean	1,874 (61.4)	
**Placenta abnormality**		
No	2,645 (86.6)	
Yes	373 (12.2)	
**Maternal history** ^c^		
No	3,011 (98.6)	
Yes	40 (1.3)	
**Delivery times**		
1	1,352 (44.3)	
2	1,146 (37.5)	
≥3	555 (18.1)	
Weight (g)	3,039 (99.5)	2238.9 ± 440.4 (730.4–4840.6)
Low birth weight	2,294 (75.5)	
Normal birth weight	745 (24.5)	
Head circumference (cm)	3,035 (99.4)	31.5 ± 5.4 (20.5–48.7)
Chest circumference (cm)	3,034 (99.3)	30.0 ± 7.9 (19.5–49.0)
Height (cm)	3,034 (99.3)	44.9 ± 4.4 (27.0–59.0)
Apgar 5 score	3,025 (99.1)	8.1 ± 1.4 (5.0–10.0)

^a^An *n* (%) is indicative of actual number of subjects equal to the total number (N), and some missing values were out of calculation; ^b^Gestational age is less than 37 weeks calculated by clinic criteria in which 7 days mean a week and if less than 5 days, it will not be calculated for a week; ^c^Maternal history indicates the occurrence of jaundice without any history of surgical trauma, infectious diseases, blood transfusion, vaccination, allergy of medicine and food, cardiovascular, cerebrovascular, lung, liver, kidney, endocrine and other important organ diseases.

### 3.2. Profiling of PM_2.5_ and PM_10_ distributions

**Figure 1:**
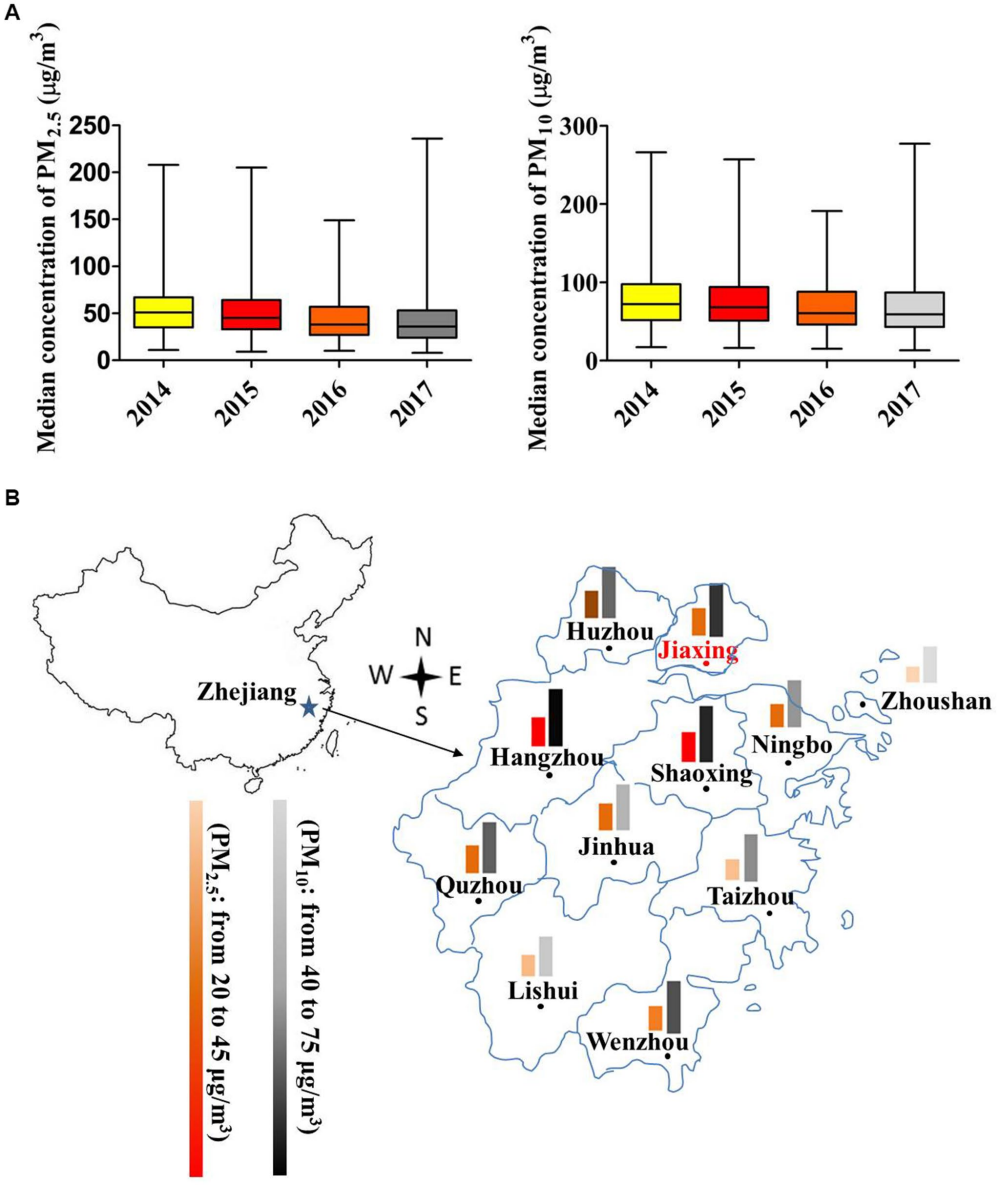
Air pollutant concentrations of Jiaxing city in 2014–2017. **(A)** The box plot of PM_2.5_ and PM_10_ concentrations in different years. The upper and lower bars were the minimum and maximum values. **(B)** PM_2.5_ and PM_10_ concentration distributions compared with near cities in 2017. The color scheme indicates the degree of pollution. ^*^ *p* < 0.05; ^**^ *p* < 0.001.

PM_10_ presented a remarkable higher concentration than PM_2.5_ (Figure 1A). The median levels (Median ± SEM) of PM_2.5_ and PM_10_ in Jiaxing presented a significant decrease in order for 2014 (51 ± 1.58 vs. 72 ± 2.24 μg/m^3^), 2015 (45 ± 1.56 vs. 68 ± 2.02 μg/m^3^), 2016 (38 ± 1.28 vs. 60.5 ± 1.78 μg/m^3^) and 2017 (36 ± 1.27 vs. 59 ± 1.71 μg/m^3^; all *p* < 0.001). Compared to other adjacent cities such as Hangzhou, Shaoxing, Ningbo, Zhoushan, Wenzhou, etc., the average levels of PM_2.5_ and PM_10_ in Jiaxing were moderate in 2017 (Figure 1B).

To acquire detailed change of PM_2.5_ and PM_10_ exposure, we categorized the exposure level via seasons during the year before birth as well as different periods of pregnancy (Table 2). The median level of PM_2.5_ was the highest in the winter (61.65 ± 0.24 μg/m^3^), followed by spring (47.34 ± 0.07 μg/m^3^), autumn (36.36 ± 0.13 μg/m^3^), and summer (30.44 ± 0.09 μg/m^3^; all *p* < 0.001). Likewise, the median level of PM_10_ in the winter (91.65 ± 0.29 μg/m^3^) was higher than that in the spring (74.01 ± 0.08 μg/m^3^), autumn (61.31 ± 0.12 μg/m^3^) and summer (51.10 ± 0.07 μg/m^3^; all *p* < 0.001). The median concentrations of PM_2.5_ and PM_10_ in the entire pregnancy were 43.37 and 68.82 μg/m^3^, respectively, with the highest level of PM_2.5_ (46.39 ± 0.26) and PM_10_ (73.98 ± 0.31; both *p* < 0.001) for the last trimester of pregnancy.

**Table 2:** Descriptives of PM_2.5_ and PM_10_ in terms of seasonal change 1 year before birth and in different time periods of pregnancy.

Category	PM_2.5_		PM_10_	
	Median ± SE	P_25_–P_75_	Median ± SE	P_25_–P_75_
**Seasons**				
Spring	47.34 ± 0.07	43.60–48.08	74.01 ± 0.08	73.24–74.38
Summer	30.44 ± 0.09	30.21–37.58	51.10 ± 0.07	50.92–56.28
Autumn	36.36 ± 0.13	35.4–47.32	61.31 ± 0.12	60.2–68.95
Winter	61.65 ± 0.24	53.49–72.43	91.65 ± 0.29	82.12–100.84
*p*-value	<0.001			
**Pregnancy time**				
First trimester	44.31 ± 0.19	37.88–51.31	70.18 ± 0.24	58.80–79.78
Second trimester	42.46 ± 0.22	34.67–50.38	65.44 ± 0.27	55.88–77.92
Last trimester	46.39 ± 0.26	33.22–53.89	73.98 ± 0.31	56.45–82.25
Entire pregnancy	43.37 ± 0.11	40.67–51.43	68.82 ± 0.10	66.88–74.86
*p*-value	<0.001			

Kruskal-Wallis *H*-test was performed between multiple groups with a further pairwise comparison, showing a statistical significance between every two groups (e.g., Spring vs. Summer).

### 3.3. Associations between PM_2.5_ and PM_10_ exposure and PTB physiological indicators

**Figure 2:**
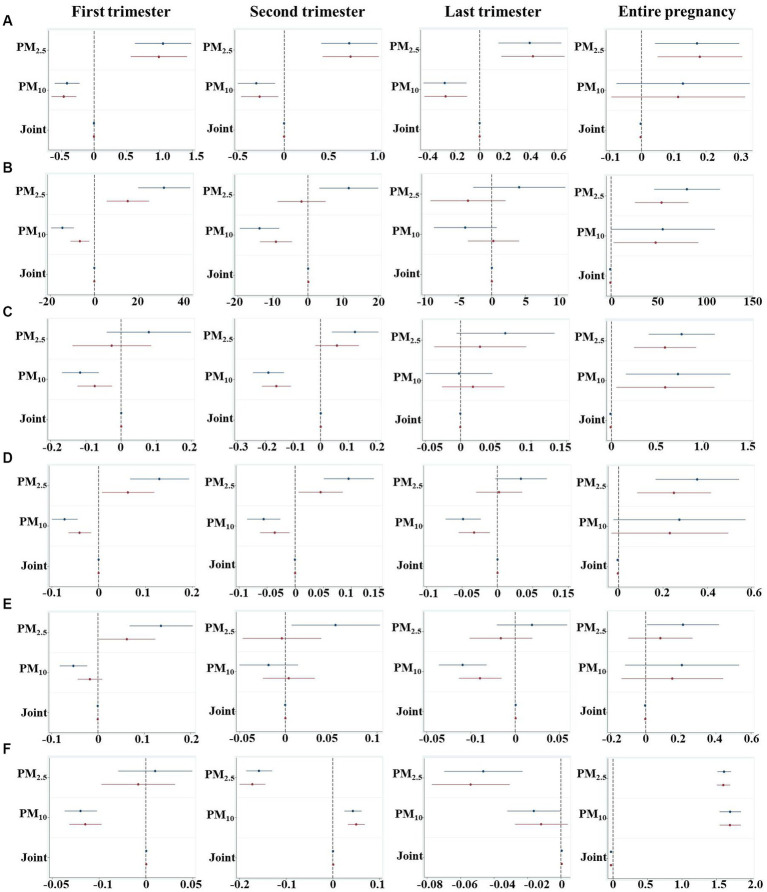
An estimated change of every 1 μg/m^3^ increment of PM_2.5_, PM_10_ and joint exposure for physiological indicators during different pregnancy periods. **(A)** Gestational age; **(B)** Weight; **(C)** Height; **(D)** Head circumference; **(E)** Chest circumference; **(F)** Apgar 5 score. The joint exposure indicated the interaction effect of PM_2.5_ and PM_10_, which was calculated by the product of PM_2.5_ and PM_10_ concentrations (PM_2.5_ * PM_10_) to evaluate the additive effect. Models for gestation were adjusted for covariates of mother education level, father smoking, maternal age, delivery times, and other models were adjusted for mother education level, father smoking, gestational age, maternal age and delivery times. The blue and red line presents unadjusted and adjusted models, respectively.

We assessed the relationships of PM_2.5_ and PM_10_ exposure in different periods of pregnancy with neonatal physiological indicators in PTB by linear regression models with adjustment for potential confounding factors or not (Figure 2). After adjusting for mother education level, father smoking, maternal age and delivery times, gestational age was positively associated with PM_2.5_ exposure (β = 0.18, 95%CI: 0.05, 0.31) for the entire pregnancy, and weakly correlated with joint PM exposure (β = −0.002, 95%CI: −0.005, −0.001; Figure 2A). However, an opposite effect was observed in the association of trimester-specific PM_2.5_ and PM_10_ exposures with gestational age whether adjusted or not.

A reduced 0.79 g of birth weight was associated with a joint exposure of PM_2.5_ and PM_10_ (95%CI: −1.371, −0.228; *p* = 0.006; Figure 2B). After stratifying the pregnancy period, we found PM_10_ was negatively correlated with birth weight whereas increased weight was associated with PM_2.5_ exposure in both the first and second trimesters, suggesting exposure to different pollutant in different window periods may bring about different effects on neonatal developmental outcomes.

In terms of the whole pregnancy, a 0.08 cm (95%CI: −0.016, −0.002; *p* = 0.008) reduction of birth height was associated with joint PM exposure, while 0.57 cm (95%CI: 0.243, 0.914; *p* = 0.001) and 0.58 cm (95%: 0.051, 1.113; *p* = 0.032) increases of height were correlated with PM_2.5_ and PM_10_ after adjustment, respectively (Figure 2C). With respect to different trimesters, decreased height was found to be associated with PM_10_ exposure in both first (95%CI: −0.168, −0.064; *p* < 0.001) and second (95%CI: −0.245, −0.134; *p* < 0.001) trimesters.

A 10 μg/m^3^ increase of joint PM exposure was associated with a 0.05 cm decrease of head circumference (95%CI: −0.084, −0.010; *p* = 0.012), whereas per unit increase of PM_2.5_ exposure was positively correlated with a 0.35 cm increment of head circumference (95%CI: 0.167, 0.543; *p* < 0.001; Figure 2D). Trimester-specific of PM_10_ exposure was correlated with lowered head circumference, while increased head circumference was found to be correlated with PM_2.5_ exposure in the first and second trimester.

Whether the chest circumference was associated with PM exposure was examined in this study. After being adjusted for confounding factors, neither PM_2.5_ and PM_10_, nor the joint exposure in the entire pregnancy was significantly correlated with chest circumference (Figure 2E). Increasing PM_2.5_ exposure in the first trimester was associated with elevated chest circumference (β = 0.06, 95%CI: 0.002, 0.123), while PM_10_ exposure in the last trimester was associated with reduced chest circumference (β = −0.04, 95%CI: −0.063, −0.015).

We further found that lowered Apgar 5 score (β = −0.03, 95%CI: −0.038, −0.025) was associated with co-exposure of PM_2.5_ and PM_10_, while increased Apgar 5 score was correlated with PM_2.5_ (β = 1.60, 95%CI: 1.506, 1.697) and PM_10_ (β = 1.69, 95%CI: 1.546, 1.850) by adjusting for confounding factors (Figure 2F). Trimester-specific exposure to PM_2.5_ or PM_10_ was found to be associated with Apgar 5 score.

### 3.4. Associations of exposures to PM_2.5_ and PM_10_ with LBW

It has been identified that LBW is correlated with ambient air pollutant exposure. In this study, we checked this association (Table 3). After adjustment for mother education level, mother smoking, father smoking, mother alcohol consumption, neonate gender, gestation, maternal age, delivery times and delivery way, LBW was found to be associated with PM_2.5_ exposure (OR = 1.37, 95%CI: 1.11, 1.68) in the entire pregnancy. No significant correlation was found for specific-trimester exposure. Given the impact of placental abnormalities, we analyzed by this stratification (Supplemental Table 1). For no placenta abnormality group, PM_2.5_ and PM_10_ exposure was somewhat correlated with LBW for the first trimester, last trimester and entire pregnancy, whereas this association disappeared after adjusting covariates of mother education level, mother smoking, father smoking, mother alcohol consumption, neonate gender, gestation, maternal age, delivery times and delivery way. There was no significant association between PM exposure and LBW for placenta abnormality group.

**Table 3:** Odds ratios (OR) of low birth weight (LBW) associated with PM_2.5_ and PM_10_ exposure during pregnancy periods were assessed by binary logistic regression models.

Pregnancy time	Crude models		Adjusted models	
	OR	95% CI	OR	95% CI
**Entire pregnancy**				
PM_2.5_	1.37	(1.11, 1.68)^**^	1.32	(1.06, 1.64)^*^
PM_10_	1.24	(0.91, 1.69)	1.25	(0.89, 1.74)
Joint	1.09	(0.98, 1.10)	1.02	(0.99, 1.07)
**First trimester**				
PM_2.5_	1.09	(1.03, 1.16)^**^	1.06	(0.99, 1.13)
PM_10_	0.97	(0.94, 0.99)^*^	0.98	(0.95, 1.01)
Joint	1.00	(0.99, 1.00)	1.00	(0.99, 1.00)
**Second trimester**				
PM_2.5_	1.00	(0.96, 1.05)	0.97	(0.92, 1.01)
PM_10_	0.97	(0.94, 1.00)	0.98	(0.95, 1.01)
Joint	1.00	(0.99, 1.00)	1.00	(0.99, 1.00)
**Last trimester**				
PM_2.5_	1.02	(0.99, 1.06)	1.00	(0.96, 1.04)
PM_10_	0.99	(0.97, 1.02)	0.99	(0.97, 1.02)
Joint	1.00	(1.00, 1.00)	1.00	(1.00, 1.00)

Covariates of mother education level, mother smoking, father smoking, mother alcohol consumption, neonate gender, gestation, maternal age, delivery times and delivery way were included in adjusted models. ^*^p < 0.05; ^**^p < 0.01.

## 4. Discussions

Environmental pollutants are associated with preterm birth, and many studies have assessed the individual PM component with PTB. In this study, we performed a retrospective study among preterm birth infants in Jiaxing city, China, and evaluated level trends of PM_2.5_ and PM_10_ in 2014–2017, and associations of PM_2.5_, PM_10_ and combined exposure with neonate developmental outcomes. We found decreasing levels for PM_2.5_ and PM_10_ in 2014–2017. This might be a result of environmental expenditures on air quality and to a certain extent the effectiveness of local environmental policies in China, as evidenced by He et al. (14) that they find a 1% increase in environmental expenditure associated with a decrease of 0.0773, 0.0125, 0.0965, and 0.0912% in the air quality index for Beijing, Taiyuan, Chongqing, and Lanzhou cities in China from the period 2007–2015. In the local, levels of PM_2.5_ and PM_10_ were moderate, which still exceed the annual standard recommended by the world health organization guidelines (WHO IT-1 for PM₂.₅ = 35 μg/m^3^; WHO IT-2 for PM₁₀ = 50 μg/m^3^) (15, 16). Therefore, mild pollution for the fine particulate matter continuously exists in Jiaxing, which should be supervised, and further studies upon the pollution sources and other pollutant constituents may be alerted. We further analyzed changes of PM_2.5_ and PM_10_ in different seasons and periods of pregnancy and found that winter or the last trimester of pregnancy was the highest for both PM_2.5_ and PM_10_. Results were similar with a previous study that they find the average PM_2.5_ concentrations of different air samples in Jiaxing during the winter and spring seasons are more severe than those in the summer and autumn, and the source may be the secondary aerosols pollution (17). Differences in pregnancy periods indicate that individuals are exposed to varying degrees of PM, and there is trimester-specific effect on PTB.

Currently, many studies regard gestational age as a confounding factor adjusted in the correlation of birth outcomes with PM exposure, few have measured this association with PM exposure. In our study, we adjust several factors related with gestational age, demonstrating a combined effect of PM_2.5_ and PM_10_ on reduced gestational age, but an opposite effect was observed in the association of trimester-specific PM_2.5_ and PM_10_ exposures with gestational age. Han et al. (18) found that trimester-specific PM_10_ exposure is positively associated with gestational age, and O_3_ exposure is linked to gestational age only in early pregnancy. Opposite to PM_10_ exposure, gestational age is reduced by 0.89 days per 10 μg/m^3^ increment in PM_2.5_ exposure and is also impacted by black carbon, organic matter and nitrate (19). Though the study result is similar to other studies, the contradictory phenomenon may be associated with the exposure level in different periods of pregnancy or specific pollutant. In our study, a combined exposure of PM_2.5_ and PM_10_ was associated with 0.79 g reductions in birth weight after adjusting for confounding factors. By stratifying the pregnancy period, PM_10_ presented a negative correlation with birth weight, while a positive correlation with the first and second trimesters for PM_2.5_. Studies have demonstrated a correlation of decreased birth weight with single ambient PM_2.5_ or PM_10_ exposure whatever using linear regression or quantile regression models for different levels of exposure (20), or satellite-based models (21). However, conclusions may vary in the association between exposures and the outcome due to different levels or the specific period time of PM exposure. Li et al. (22) observe that ambient air pollutant concentrations during pregnancy are not associated with reduced term birth weight, but PM_2.5_ concentration in the 6th gestational month is associated with a −20.4 g reduction in term birth weight among Hispanic women. Similar to our result, a prior study from North Carolina indicates positive associations of PM_2.5_ and O_3_ exposure with term low birth weight (10). Though the reason causing this side effect on birth weight is not definitely clear, some biological mechanisms have been identified that PM exposure induces sustained oxidative stress and inflammation. Changes in expressions of IL-17 and EGF are linked with air pollution-associated shifts in birth weight (23). Epigenetic modifications, for example, DNA methylation, have been proved to affect several biological mechanisms with marked effects during susceptible life stages such as pregnancy (24). Therefore, apart from a single pollutant of PM_2.5_ or PM_10_, multiple sources of exposure should be considered during *in utero* development.

With respect to birth height, a combined exposure of PM_2.5_ and PM_10_ in the whole pregnancy was correlated with 0.08 cm decrease in birth height, while PM_2.5_ and PM_10_ showed an opposite effect, with PM_10_ levels in the first and second trimester associated with decreased height. In this study, PM_2.5_ and PM_10_ levels were different in different seasons and periods of pregnancy, which may have different restrictions on birth height. Spears et al. assesses the association between early-life ambient PM_2.5_ and subsequent height-for-age, showing a 0.24 cm height deficit among an average 5 year old girl, and exposure in the first few months of life is significantly associated with child height deficits, indicating that exposure to PM_2.5_ at different developmental stages could produce reduced or null effect (25). Another study from Poland presents that mean height decreases with growing PM_10_ and PM_2.5_ levels, and significant differences are observed both in absolute and relative height (expressed as percentage of mean stature of both parents) due to levels in place of residence during childhood and adolescence (26). Our results add to the evidence regarding the adverse joint effect of PM_10_ and PM_2.5_ on fetus height. In this study, we found a 0.05 cm decrease in head circumference associated with a joint exposure, while PM_2.5_ presented an opposite effect. Increased PM_2.5_ exposure is significantly associated with a 0.04 cm reduction in head circumference, which might be associated with aberrant changes in DNA methylation profile of placenta genome leading to disordered energy metabolism and immune response (27). However, one study from Tanzania shows that PM_2.5_ exposure is not significantly associated with head circumference, though they enroll 239 women in their study (28). Therefore, results may distinguish between studies as the exposure level is different as well as the sensitive time window during *in utero* development. In addition, PM_10_ is also an influencing factor contributing to the reduced head circumference. PM_10_ exposure can induce sustained oxidative stress and inflammation and causes autonomic nervous system activation (29). So a combined exposure should be alerted.

Moreover, we checked the association of chest circumference with PM exposure. For the whole pregnancy, no correlation of chest circumference with PM exposure was found, while increased PM_2.5_ in the first trimester was positively correlated with chest circumference, and PM_10_ in the last trimester was negatively correlated with chest circumference. Other studies find that PM exposure during the whole pregnancy exhibits no correlation with chest circumference (30, 31). This suggests a potential influence of different sources of exposure and exposure time during pregnancy on chest circumference. Apgar 5 score has been a useful indicator providing prognostic information about neonatal survival among preterm infants (32). In our study, lowered Apgar 5 score was associated with co-exposure of PM_2.5_ and PM_10_, while single PM_2.5_ or PM_10_ exposure showed difference. Increased Apgar 1 score has been reported to be associated with trimester-specific exposure to soil dust (33). This indicates that abnormal Apgar score may be associated with PM exposure levels in different trimesters. Most studies have shown the correlation of PM_2.5_ exposure with LBW (34). In our study, after adjusting the covariates, PM_2.5_ was associated with LBW, while no trimester-specific effect was found. Studies have found that a higher short-term exposure of PM_2.5_ is associated with LBW in developmental children (35). A recent study on large scale from Spain demonstrates that PM_10_ (OR = 1.104) and NO_2_ (OR = 1.091) during the entire pregnancy, rather than PM_2.5_ (not measured), was associated with elevated risk of LBW (36). PM_2.5_ exposure in different gestational week is associated with adverse birth outcomes in infants (37). By stratifying placental abnormalities, though PM_2.5_ or PM_10_ exposure was somewhat correlated with LBW for the first trimester, last trimester and entire pregnancy, we found no associations between PM exposure and LBW after adjusting confounding factors. These results suggest that LBW is a result of complex exposure and biological mechanism during pregnancy.

There are also some limitations in this study. First, the components of air pollutants are complex, and there are other air pollutants or factors that may influence preterm birth such as CO, SO_2_, NO_2_, O_3_, temperature and humidity (38). There are also other influencing factors like socioeconomic factors of single parent families, maternal occupation and family income, as well as nutritional and medical factors (13, 39). As this information is unavailable, we could not include the impact of these factors when assessing the associations of PM exposure and preterm birth indicators. Secondly, exposure levels of PM_2.5_ and PM_10_ are incompletely accurate representing the maternal and infant exposures, which may bring some biases for the assessment of exposure-response, particular for the varying effects of joint exposure and the individual exposure of PM_2.5_ or PM_10_. The interaction of PM_2.5_ and PM_10_ may also have an impact as they are highly correlated, and the weight of the two pollutants may be considered (40). Thirdly, this is a retrospective study for PTB, and we did not recruit the healthy babies to overall evaluate the prevalence of PTB and the adverse birth outcomes. Again, there are many aspects of adverse birth outcomes, including the physical examinations, diseases or lesions, such as inflammation, malformation of the cardiovascular system, respiratory distress, the application of drug therapy, and small changes in blood biochemical indicators of newborns. Such information might be considered to find more detailed health problems linking with local air pollutants. The strength of the evidence is not strong enough to reflect the newborn development, especially for the impact of development in their later life. Whatever from the study design, real-time exposure levels or susceptible population, future studies should be continuously encouraged to evaluate the relationship between exposure of different air pollutants *in utero* and health outcomes.

## 5. Conclusion

In total, we find mild contamination of fine particulate matter in atmospheric environment in Jiaxing, with season-specific changes. This alteration increases the risk of reduced neonatal development *in utero*, especially the combined exposure of PM_2.5_ and PM_10_, whereas the results are sometimes opposite under individual exposure of PM_2.5_ or PM_10_ at different pregnancy periods, suggesting a time-window and pollutant-specific effect. Findings from this study could provide clues for the policy maker to take certain measures to control the local air pollution. Future studies are needed to focus on the health assessment for such vulnerable populations impacted by fine particulate matter.

## Data availability statement

The original contributions presented in the study are included in the article/Supplementary material, further inquiries can be directed to the corresponding authors.

## Author contributions

BH: data collection and writing the draft. JT: data input management. GX: data supervision and resource support. DS and HH: data resources and collection. JL, HC, JC, LZ, SC, and BS: data collection and input. LJ: conceptualize the idea and writing. LX: conceptualize the idea, statistical analysis, funding acquisition, and writing and editing. All authors contributed to the article and approved the submitted version.

## Funding

This work was financially supported by National Natural Science Foundation of China (22206059) and Medical Health Science and Technology Project of Zhejiang Provincial Health Commission under Grant (2023RC101).

## Conflict of interest

The authors declare that the research was conducted in the absence of any commercial or financial relationships that could be construed as a potential conflict of interest.

## Publisher’s note

All claims expressed in this article are solely those of the authors and do not necessarily represent those of their affiliated organizations, or those of the publisher, the editors and the reviewers. Any product that may be evaluated in this article, or claim that may be made by its manufacturer, is not guaranteed or endorsed by the publisher.

## Footnotes

^1^ http://www.cnemc.cn/
