# Supplementary material for: Combined exposure to PM2.5 and PM10 in reductions of physiological development among preterm birth: a retrospective study from 2014 to 2017 in China
Source: Front Public Health. 2023 Jul 26;11:1146283. doi: 10.3389/fpubh.2023.1146283 (PMC10410271; doi:10.3389/fpubh.2023.1146283)
Supplement: Supplementary file 1 [file Table_1.doc]

**Supplementary Materials**

**Combined exposure to PM_2.5_ and PM_10_** **in reductions of physiological development among preterm birth: A retrospective study from 2014-2017 in China**

Bo Hu^a,b^, Jie Tang ^a,b^, Guangtao Xu^a^, Dongliang Shao^c^, Huafei Huang^c^, Jintong Li^a^, Huan Chen^a^, Jie Chen^a^, Liangjin Zhu^a^, Shipiao Chen^a^, Bin Shen^a^, Limin Jin^b#^, Long Xu^a#^

^a^ Department of Preventive Medicine, Forensic and Pathology Laboratory, Institute of Forensic Science, College of Medicine, Jiaxing University, Jiaxing, 314001, ZJ, China

^b^ Department of Pathology and Key-Innovative Discipline Molecular Diagnostics, Jiaxing Hospital of Traditional Chinese Medicine, Jiaxing University, Jiaxing, 314001, ZJ, China

^c^ Department of Neonatal Intensive Care Unit, Jiaxing Maternity and Child Health Care Hospital, Jiaxing University, Jiaxing 314001, ZJ, China

**Supplemental Table 1.** Odds ratios (OR) of low birth weight (LBW) associated with PM_2.5_ and PM_10_ exposure during pregnancy periods stratified by placenta abnormality.

|  |  | Crude models | |  | Adjusted models | |
| --- | --- | --- | --- | --- | --- | --- |
|  |  | OR | 95% CI |  | OR | 95% CI |
| **Placenta abnormality** |  |  |  |  |  |  |
| Entire pregnancy |  |  |  |  |  |  |
| PM_2.5_ |  | 1.05 | (0.73, 1.37) |  | 1.03 | (0.68, 1.38) |
| PM_10_ |  | 0.96 | (0.66, 1.26) |  | 0.95 | (0.62, 1.28) |
| Joint |  | 1.00 | (1.00, 1.00) |  | 1.00 | (1.00, 1.00) |
| First trimester |  |  |  |  |  |  |
| PM_2.5_ |  | 0.97 | (0.88, 1.06) |  | 0.96 | (0.87, 1.05) |
| PM_10_ |  | 1.04 | (0.95, 1.13) |  | 1.05 | (0.95, 1.15) |
| Joint |  | 1.00 | (1.00, 1.00) |  | 1.00 | (1.00, 1.00) |
| Second trimester |  |  |  |  |  |  |
| PM_2.5_ |  | 1.04 | (0.91, 1.17) |  | 0.98 | (0.84, 1.12) |
| PM_10_ |  | 1.01 | (0.92, 1.10) |  | 1.06 | (0.95, 1.17) |
| Joint |  | 1.00 | (1.00, 1.00) |  | 1.00 | (1.00, 1.00) |
| Last trimester |  |  |  |  |  |  |
| PM_2.5_ |  | 1.05 | (0.96, 1.14) |  | 1.01 | (0.91, 1.12) |
| PM_10_ |  | 0.97 | (0.89, 1.05) |  | 0.98 | (0.89, 1.08) |
| Joint |  | 1.00 | (1.00, 1.00) |  | 1.00 | (1.00, 1.00) |
| **No placenta abnormality** |  |  |  |  |  |  |
| Entire pregnancy |  |  |  |  |  |  |
| PM_2.5_ |  | 1.14 | (1.05, 1.23)** |  | 1.08 | (0.99, 1.17) |
| PM_10_ |  | 0.89 | (0.81, 0.96)** |  | 0.93 | (0.85, 1.01) |
| Joint |  | 1.00 | (1.00, 1.00) |  | 1.00 | (1.00, 1.00) |
| First trimester |  |  |  |  |  |  |
| PM_2.5_ |  | 0.95 | (0.92, 0.98)* |  | 0.98 | (0.94, 1.02) |
| PM_10_ |  | 1.05 | (1.01, 1.09)** |  | 1.03 | (0.99, 1.07) |
| Joint |  | 1.00 | (1.00, 1.00) |  | 1.00 | (1.00, 1.00) |
| Second trimester |  |  |  |  |  |  |
| PM_2.5_ |  | 1.00 | (0.97, 1.05) |  | 0.98 | (0.93, 1.03) |
| PM_10_ |  | 0.97 | (0.94, 1.00) |  | 0.97 | (0.94, 1.00) |
| Joint |  | 1.00 | (1.00, 1.00) |  | 1.00 | (1.00, 1.00) |
| Last trimester |  |  |  |  |  |  |
| PM_2.5_ |  | 1.02 | (0.99, 1.05)* |  | 1.01 | (0.99, 1.03) |
| PM_10_ |  | 0.99 | (0.97, 1.01) |  | 0.99 | (0.97, 1.01) |
| Joint |  | 1.00 | (1.00, 1.00) |  | 1.00 | (1.00, 1.00) |

Note: Covariates of mother education level, mother smoking, father smoking, mother alcohol consumption, neonate gender, gestation, maternal age, delivery times and delivery way were included in adjusted models. ^*^ *p* < 0.05; ^**^ *p* < 0.01.
